# Poly[[tri-μ-aqua-do­deca­aqua­tris­(μ_3_-1-hy­droxy­ethyl­idene-1,1-di­phospho­nato)tricalcium(II)tripalladium(II)] penta­hydrate]

**DOI:** 10.1107/S1600536814015189

**Published:** 2014-07-05

**Authors:** Irina P. Kutsenko, Alexandra N. Kozachkova, Natalia V. Tsaryk, Vasily I. Pekhnyo, Julia A. Rusanova

**Affiliations:** a V.I. Vernadsky Institute of General and Inorganic Chemistry of Ukraine, National Academy of Sciences, Kiev-142, prospekt Akademika Palladina 32/34, Ukraine; b Taras Shevchenko National University, Department of Inorganic Chemistry, Volodymyrska str. 64/13, 01601 Kyiv, Ukraine

**Keywords:** crystal structure

## Abstract

The asymmetric unit of the title compound, {[CaPd{CH_3_OHC(PO_3_)_2_}(H_2_O)_5_]·5/3H_2_O}_*n*_, consists of one half of the complex [Pd{CH_3_OHC(PO_3_)_2_}]^2−^ anion (point group symmetry *m*..), one Ca^2+^ cation [site symmetry (.2.)] that is surrounded by three water mol­ecules (one of which is on the same rotation axis) and by three disordered lattice water mol­ecules. The anions form a trinuclear metallocycle around a crystallographic threefold rotation axis. The cations are related by a twofold rotation axis to form a [Ca_2_(H_2_O)_10_]^2+^ dimer. The slightly distorted square-planar coordination environment of the Pd^II^ atoms in the complex anions is formed by O atoms of the bidentate chelating phospho­nate groups of the 1-hy­droxy­ethyl­idene-1,1-di­phospho­nate ligands. In the crystal, cations are bound to anions through —Ca—O—P—O— bonds, as well as through O—H⋯O hydrogen bonds, resulting in a three-dimensional polymer. The structure is completed by five disordered solvent mol­ecules localized in cavities within the framework.

## Related literature   

For background to di­phospho­nic acids see: Zhang *et al.* (2007 ▶); Szabo *et al.* (2002 ▶); Matczak-Jon & Videnova-Adrabinska (2005 ▶). For background to the anti­tumor activity of palladium(II) complexes, see: Juribašiċ *et al.* (2011 ▶); Curic *et al.* (1996 ▶); Abu-Surrah *et al.* (2008 ▶); Ruiz *et al.* (2005 ▶, 2006 ▶); Tušek-Božiċ *et al.* (2008 ▶). For the structures of related complexes, see: Babaryk *et al.* (2012 ▶); Hammerl *et al.* (2002 ▶); Müller (1972 ▶). 
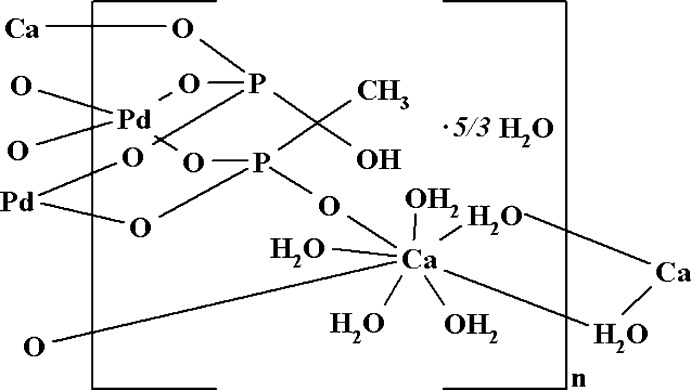


## Experimental   

### 

#### Crystal data  

[CaPd(C_2_H_4_O_7_P_2_)(H_2_O)_5_]·1.67H_2_O
*M* *_r_* = 468.58
Hexagonal, 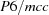

*a* = 15.9731 (3) Å
*c* = 18.4149 (4) Å
*V* = 4068.91 (14) Å^3^
*Z* = 12
Mo *K*α radiation
μ = 2.05 mm^−1^
*T* = 296 K
0.39 × 0.07 × 0.06 mm

#### Data collection  

Bruker APEXII CCD area-detector diffractometer
Absorption correction: multi-scan (*SADABS*; Sheldrick, 2009 ▶) *T*_min_ = 0.502, *T*_max_ = 0.887
37638 measured reflections
1774 independent reflections
1410 reflections with *I* > 2σ(*I*)
*R*_int_ = 0.082

#### Refinement  

*R*[*F* ^2^ > 2σ(*F* ^2^)] = 0.031
*wR*(*F* ^2^) = 0.071
*S* = 1.08
1774 reflections
116 parameters
28 restraints
H atoms treated by a mixture of independent and constrained refinement
Δρ_max_ = 0.56 e Å^−3^
Δρ_min_ = −0.57 e Å^−3^

### 

Data collection: *APEX2* (Bruker, 2007 ▶); cell refinement: *SAINT* (Bruker, 2007 ▶); data reduction: *SAINT*; program(s) used to solve structure: *SHELXTL* (Sheldrick, 2008 ▶); program(s) used to refine structure: *SHELXTL*; molecular graphics: *SHELXTL*; software used to prepare material for publication: *publCIF* (Westrip, 2010 ▶).

## Supplementary Material

Crystal structure: contains datablock(s) I, New_Global_Publ_Block. DOI: 10.1107/S1600536814015189/br2239sup1.cif

Structure factors: contains datablock(s) I. DOI: 10.1107/S1600536814015189/br2239Isup2.hkl

CCDC reference: 1010912

Additional supporting information:  crystallographic information; 3D view; checkCIF report

## Figures and Tables

**Table 1:** Hydrogen-bond geometry (Å, °)

*D*—H⋯*A*	*D*—H	H⋯*A*	*D*⋯*A*	*D*—H⋯*A*
O5—H1⋯O1^i^	0.84 (1)	1.95 (2)	2.756 (3)	160 (4)
O5—H2⋯O5^ii^	0.79 (2)	2.07 (2)	2.799 (5)	155 (4)
O6—H3⋯O2^iii^	0.82 (2)	1.87 (2)	2.685 (3)	170 (4)
O7—H4⋯O3^i^	0.82 (2)	2.07 (2)	2.865 (4)	166 (4)
O4*A*—H4*A*⋯O8	0.82	1.99	2.731 (16)	150

Symmetry codes: (i) 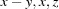; (ii) 
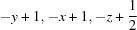
; (iii) 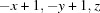.

**Fig. 1.**
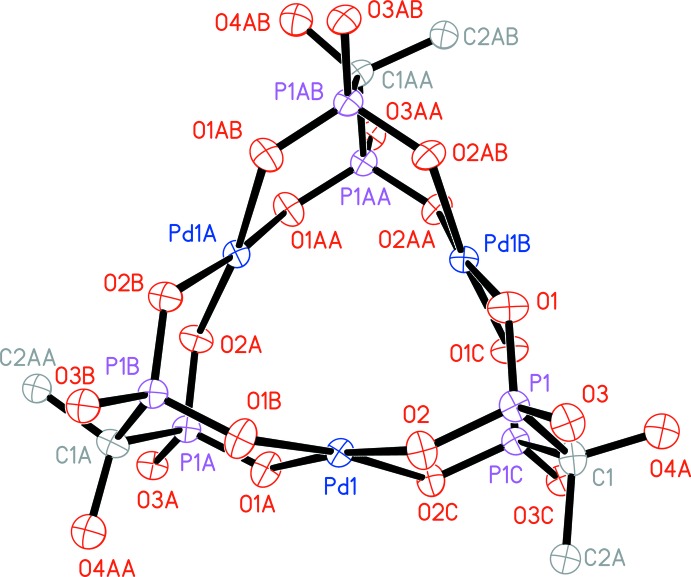
View of the anionic part of [Pd3{O3PC(OH)(CH3)PO3}3]6- unit showing 50% probability displacement ellipsoids for the non-hydrogen atoms.

**Fig. 2.**
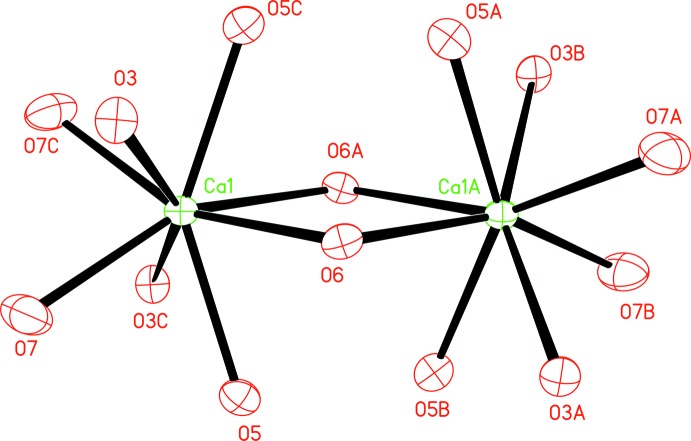
View of the cationic part of [(4H2O)O2Ca(2H2O)CaO2(4H2O)]2+ fragments showing 50% probability displacement ellipsoids for the non-hydrogen atoms.

**Fig. 3.**
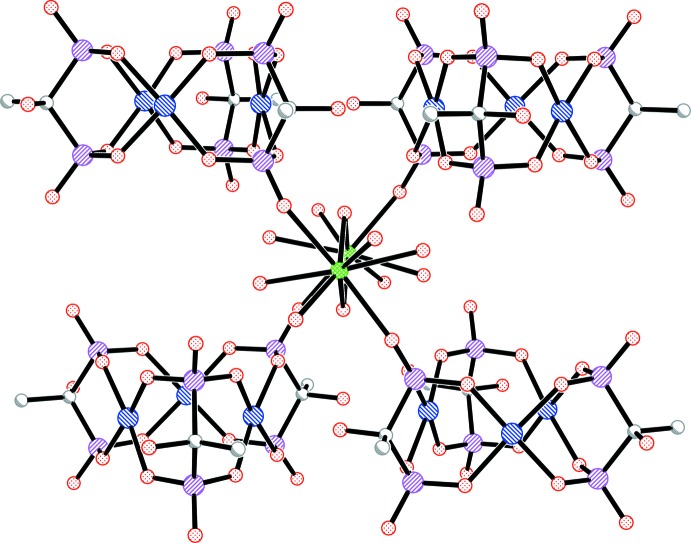
Linking of cations to anions of adjacent layers in crystal packing.

**Fig. 4.**
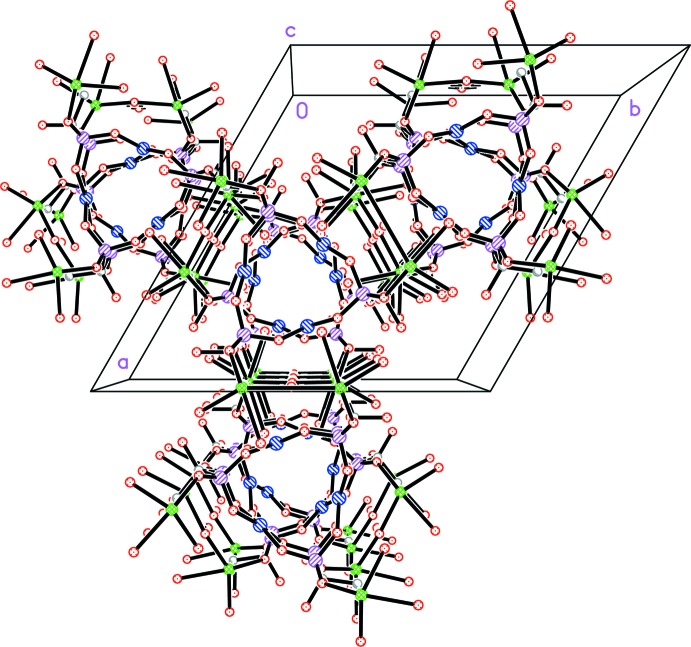
Crystal packing of title compound, in a projection along the c axis. Solvate molecules as well as hydrogen bonds are omitted for clarity.

## supplementary crystallographic information

### S1. Comment

During the last decade, there has been a growing interest in the study of organic diphosphonic acids owing to their potentially very powerful chelating properties used in metal extractions and are tested by the pharmaceutical industry for use as efficient drugs preventing calcification and inhibiting bone resorption (Matczak-Jon & Videnova-Adrabinska, 2005). Diphosphonic acids and their metal complexes are used in the treatment of Pagets disease, osteoporosis and tumoral osteolysis (Szabo *et al.*, 2002). Also in the last years, there has been a surge of interest in palladium complexes as a prospective antitumor preparation (Abu-Surrah *et al.*, 2008, Curic *et al.*, 1996).

The title compound crystallized in centric space group P6/mcc. The square-planar environment of palladium atoms in the complex anion [Pd_3_{CH_3_OHC(PO_3_)_2_}_3_]^6-^is formed by coordination of the oxygen atoms of the chelating phosphonic groups of the ligand. By crystallographic threefold rotation axis it completed to trinuclear species with equilateral triangle geometry (Fig.1). Palladium atoms slightly deviate from the oxygen mean-planes towards the triangle center by 0.12 Å. The range of Pd – O bond distances of 2,006 (2) – 2,010 (2)Å as well *cis* O–Pd–O angles ranging from 85.78 (9)° to 92.91 (13)° are in a good agreement with literature values (Babaryk *et al.*, 2012 and references therein). CH_3_ and OH groups of the HEDP are statistically disordered over two positions with equal occupation numbers.

As it shown on Fig. 2, each Ca atom of the complex binuclear cation is surrounded by eight oxygen atoms (six from water molecules, comprising two bridging ones, and two from phosphonic groups of the trinuclear clusters) in the form of a slightly distorted, bi-capped trigonal prism. (Hammerl *et al.*, 2002). The Ca – O bond distances were observed within the range of 2,416 (3) – 2,538 (3)Å. Calcium coordinated by eight water molecules is well known in the literature, and the distances of the bridging and nonbridging oxygen atoms found here agree well with the previously reported values (Müller *et al.*, 1972). Each binuclear cation linked to four trinuclear anions of adjacent layers through -Ca-O-P-O- bonds (Fig. 3) as well thought O-H···O hydrogen bonds. Moreover, each trinuclear anion is linked to six binuclear cations. In the crystal packing cations and anions stacked along the *c* axis into columns where layer of cations alternates with layer of anions. Resulting layered 3d polymer structure (Fig.4) completed by five disordered solvent water molecules which are located in cavities. The Ca – O and P-O bond distances (2.507 (2)Å and 1.498 (2)Å respectively) as well as D···A distances ranging from 2.756 (3)Å to 2.865 (4)Å are in a good agreement with literature values. Oxygen atom of one of the water solvent molecules O8 is statistically disordered over two positions and linked to cation thought CH_3_-C-OH···O8 hydrogen bonds with D···A distance about 2.731 (16) Å. Another one water molecule oxygen atom O9 is situated at the origin (0, 0, 0) with 1/12 multiplicity. Remaining 3 oxygen atoms O10 of the water molecules are disordered around 6-fold rotation axis with centre of symmetry over 12 positions with an occupation number of 1/4. Considering this reasonable H-atom positions of the disordered solvent water molecules were not established

### S2. Experimental

A solution of AgNO_3_ (0.3398 g, 2.0 mmol) in H_2_O (5 ml) was added to a solution of PdCl_2_ (0.0885 g, 0.5 mmol) in hydrochloric acid (0.1*M*, 10 ml) and the resulting solution stirred at 276 K for 30 min under protection from light until AgCl was precipitated and filtered off. Hydroxyethylidenediphosphonic acid (0.112 g, 0.5 mmol) and CaCO_3_ (0.05 g, 0.5 mmol) were added to filtrate. The resulting solution was stirred for 1 h at 276 - 277 K and left staying overnight at room temperature. The solvent was removed from resulting reaction mixture under reduced pressure leaving an yellow solid, which was washed twice with methanol and diethyl ether and dried under vacuum. Yellow rectangular crystals of the title compound suitable for crystallographic study were produced by slow evaporation of a water solution at room temperature.

### S3. Refinement

The structure was solved by the direct method. H atoms of methyl groups were placed at calculated positions and treated as riding on the parent atoms, with *U*_iso_(H) = 1.5 *U*_eq_(C). H atoms near of the O4A and O4B atoms, and H atoms of water molecule were located in a difference Fourier map and further refined with SADI instruction to the restraint that they should be equal within about 0.02 Å and *U*_iso_(H) = 1.5Ueq(O). The OH and CH_3_ groups near C(1) carbon atom are disordered over two position with occupancy factor 0.25 and 0.25 respectively. Oxygen atoms of the water solvent molecules O8, O9, O10 were refined isotropically- O8 is statistically disordered with occupancy 1/2 and it position depends on the disorder of the above mentioned OH group; O9 is situated at the origin (0, 0, 0) with 1/12 multiplicity; three oxygen atoms O10 are disordered around 6-fold rotation axis with centre of symmetry over 12 positions with an occupation number of 1/4. Considering this reasonable H-atom positions of the disordered solvent water molecules were not established.

### Crystal data

**Table:**

[CaPd(C_2_H_4_O_7_P_2_)(H_2_O)_5_]·1.67H_2_O	*F*(000) = 2816
*M**_r_* = 468.58	*D*_x_ = 2.295 Mg m^−^^3^
Hexagonal, *P*6/*m**c**c*	Mo *K*α radiation, λ = 0.71073 Å
Hall symbol: -P 6 2c	µ = 2.05 mm^−^^1^
*a* = 15.9731 (3) Å	*T* = 296 K
*c* = 18.4149 (4) Å	Rectangular, yellow
*V* = 4068.91 (14) Å^3^	0.39 × 0.07 × 0.06 mm
*Z* = 12	

### Data collection

**Table:**

Bruker APEXII CCD area-detector diffractometer	1774 independent reflections
Radiation source: fine-focus sealed tube	1410 reflections with *I* > 2σ(*I*)
Graphite monochromator	*R*_int_ = 0.082
phi and ω scans	θ_max_ = 28.4°, θ_min_ = 2.6°
Absorption correction: multi-scan (*SADABS*; Sheldrick, 2009)	*h* = −21→21
*T*_min_ = 0.502, *T*_max_ = 0.887	*k* = −21→18
37638 measured reflections	*l* = −22→24

### Refinement

**Table:**

Refinement on *F*^2^	Primary atom site location: structure-invariant direct methods
Least-squares matrix: full	Secondary atom site location: difference Fourier map
*R*[*F*^2^ > 2σ(*F*^2^)] = 0.031	Hydrogen site location: inferred from neighbouring sites
*wR*(*F*^2^) = 0.071	H atoms treated by a mixture of independent and constrained refinement
*S* = 1.08	*w* = 1/[σ^2^(*F*_o_^2^) + (0.0249*P*)^2^ + 11.4857*P*] where *P* = (*F*_o_^2^ + 2*F*_c_^2^)/3
1774 reflections	(Δ/σ)_max_ = 0.013
116 parameters	Δρ_max_ = 0.56 e Å^−^^3^
28 restraints	Δρ_min_ = −0.57 e Å^−^^3^

### Special details

**Table:**

Geometry. All e.s.d.'s (except the e.s.d. in the dihedral angle between two l.s. planes) are estimated using the full covariance matrix. The cell e.s.d.'s are taken into account individually in the estimation of e.s.d.'s in distances, angles and torsion angles; correlations between e.s.d.'s in cell parameters are only used when they are defined by crystal symmetry. An approximate (isotropic) treatment of cell e.s.d.'s is used for estimating e.s.d.'s involving l.s. planes.
Refinement. Refinement of *F*^2^ against ALL reflections. The weighted *R*-factor *wR* and goodness of fit *S* are based on *F*^2^, conventional *R*-factors *R* are based on *F*, with *F* set to zero for negative *F*^2^. The threshold expression of *F*^2^ > σ(*F*^2^) is used only for calculating *R*-factors(gt) *etc*. and is not relevant to the choice of reflections for refinement. *R*-factors based on *F*^2^ are statistically about twice as large as those based on *F*, and *R*- factors based on ALL data will be even larger.

### Fractional atomic coordinates and isotropic or equivalent isotropic displacement parameters (Å^2^)

**Table:**

	*x*	*y*	*z*	*U*_iso_*/*U*_eq_	Occ. (<1)
Pd1	0.63688 (3)	0.44960 (3)	0.0000	0.01659 (11)	
Ca1	0.37002 (6)	0.37002 (6)	0.2500	0.0182 (2)	
P1	0.45030 (6)	0.28992 (6)	0.08236 (4)	0.01779 (18)	
O1	0.48149 (18)	0.21370 (18)	0.08028 (12)	0.0246 (5)	
O2	0.53726 (17)	0.39316 (17)	0.07897 (12)	0.0223 (5)	
O3	0.38931 (18)	0.27739 (18)	0.14777 (12)	0.0218 (5)	
O5	0.33373 (19)	0.50409 (19)	0.22123 (13)	0.0243 (5)	
H1	0.327 (3)	0.507 (3)	0.1764 (6)	0.037*	
H2	0.367 (2)	0.5554 (18)	0.2384 (19)	0.037*	
O6	0.5000	0.5000	0.17187 (18)	0.0221 (7)	
H3	0.484 (3)	0.526 (3)	0.1407 (15)	0.033*	
O7	0.2157 (2)	0.2933 (2)	0.18857 (17)	0.0395 (7)	
H4	0.182 (3)	0.318 (3)	0.184 (2)	0.059*	
H5	0.238 (3)	0.291 (4)	0.1493 (16)	0.059*	
C1	0.3787 (3)	0.2740 (3)	0.0000	0.0224 (10)	
O4A	0.2883 (5)	0.1836 (5)	0.0000	0.0311 (19)*	0.50
H4A	0.253 (4)	0.185 (4)	−0.031 (6)	0.047*	0.25
C2A	0.3627 (7)	0.3633 (5)	0.0000	0.023 (2)*	0.50
H21	0.3251	0.3600	−0.0416	0.034*	0.25
H22	0.4242	0.4218	−0.0018	0.034*	0.50
H23	0.3291	0.3626	0.0434	0.034*	0.25
C2B	0.3085 (7)	0.1626 (5)	0.0000	0.019 (2)*	0.50
H24	0.2910	0.1403	0.0491	0.029*	0.25
H25	0.3399	0.1310	−0.0219	0.029*	0.25
H26	0.2515	0.1478	−0.0271	0.029*	0.25
O4B	0.3386 (6)	0.3370 (6)	0.0000	0.029*	0.50
H4B	0.298 (7)	0.321 (6)	−0.033 (5)	0.029*	0.25
O8	0.1458 (11)	0.1163 (12)	−0.1026 (9)	0.169 (6)*	0.50
O9	0.0000	0.0000	0.0000	0.40 (3)*	
O10	0.108 (2)	0.079 (3)	−0.2084 (13)	0.184 (14)*	0.25

### Atomic displacement parameters (Å^2^)

**Table:**

	*U*^11^	*U*^22^	*U*^33^	*U*^12^	*U*^13^	*U*^23^
Pd1	0.01985 (19)	0.0223 (2)	0.00785 (16)	0.01075 (16)	0.000	0.000
Ca1	0.0199 (3)	0.0199 (3)	0.0176 (4)	0.0119 (4)	−0.00021 (18)	0.00021 (18)
P1	0.0220 (4)	0.0215 (4)	0.0089 (4)	0.0101 (4)	0.0018 (3)	−0.0004 (3)
O1	0.0386 (15)	0.0328 (14)	0.0105 (11)	0.0240 (12)	0.0048 (10)	0.0016 (10)
O2	0.0237 (12)	0.0255 (12)	0.0119 (11)	0.0080 (10)	0.0043 (10)	−0.0021 (9)
O3	0.0261 (13)	0.0265 (13)	0.0106 (11)	0.0116 (11)	0.0045 (9)	−0.0005 (10)
O5	0.0261 (14)	0.0318 (14)	0.0165 (12)	0.0155 (12)	−0.0011 (10)	0.0024 (10)
O6	0.0280 (19)	0.0228 (18)	0.0194 (17)	0.0156 (15)	0.000	0.000
O7	0.0380 (17)	0.0476 (18)	0.0439 (18)	0.0297 (15)	−0.0130 (14)	−0.0102 (15)
C1	0.021 (2)	0.025 (3)	0.016 (2)	0.007 (2)	0.000	0.000

### Geometric parameters (Å, º)

**Table:**

Pd1—O2^i^	2.006 (2)	Ca1—O5^iv^	2.538 (3)
Pd1—O2	2.006 (2)	Ca1—Ca1^vi^	4.1524 (18)
Pd1—O1^ii^	2.010 (2)	P1—O3	1.498 (2)
Pd1—O1^iii^	2.010 (2)	P1—O1	1.529 (2)
Ca1—O7	2.416 (3)	P1—O2	1.537 (2)
Ca1—O7^iv^	2.416 (3)	P1—C1	1.839 (3)
Ca1—O3	2.507 (2)	C1—O4B	1.438 (6)
Ca1—O3^iv^	2.507 (2)	C1—O4A	1.444 (6)
Ca1—O6^v^	2.526 (2)	C1—C2B	1.559 (6)
Ca1—O6	2.526 (2)	C1—C2A	1.569 (6)
Ca1—O5	2.538 (3)	C1—P1^i^	1.839 (3)
			
O2^i^—Pd1—O2	92.91 (13)	O6^v^—Ca1—O5^iv^	68.18 (6)
O2^i^—Pd1—O1^ii^	85.78 (9)	O6—Ca1—O5^iv^	82.30 (7)
O2—Pd1—O1^ii^	173.09 (10)	O5—Ca1—O5^iv^	144.16 (13)
O2^i^—Pd1—O1^iii^	173.09 (10)	O7—Ca1—Ca1^vi^	139.79 (8)
O2—Pd1—O1^iii^	85.78 (9)	O7^iv^—Ca1—Ca1^vi^	139.79 (8)
O1^ii^—Pd1—O1^iii^	94.70 (14)	O3—Ca1—Ca1^vi^	103.51 (6)
O7—Ca1—O7^iv^	80.42 (16)	O3^iv^—Ca1—Ca1^vi^	103.51 (6)
O7—Ca1—O3	75.19 (9)	O3—P1—O1	111.26 (14)
O7^iv^—Ca1—O3	84.19 (10)	O3—P1—O2	110.76 (13)
O7—Ca1—O3^iv^	84.19 (10)	O1—P1—O2	111.95 (14)
O7^iv^—Ca1—O3^iv^	75.19 (9)	O3—P1—C1	109.08 (15)
O3—Ca1—O3^iv^	152.97 (12)	O1—P1—C1	107.27 (16)
O7—Ca1—O6^v^	153.43 (7)	O2—P1—C1	106.30 (16)
O7^iv^—Ca1—O6^v^	111.16 (10)	P1—O1—Pd1^vii^	128.04 (14)
O3—Ca1—O6^v^	128.30 (7)	P1—O2—Pd1	126.86 (14)
O3^iv^—Ca1—O6^v^	76.37 (7)	P1—O3—Ca1	142.04 (15)
O7—Ca1—O6	111.16 (10)	Ca1^vi^—O6—Ca1	110.56 (13)
O7^iv^—Ca1—O6	153.43 (7)	O4B—C1—O4A	97.3 (6)
O3—Ca1—O6	76.37 (7)	O4B—C1—C2B	118.7 (6)
O3^iv^—Ca1—O6	128.30 (7)	O4A—C1—C2A	111.9 (6)
O6^v^—Ca1—O6	69.44 (13)	C2B—C1—C2A	133.4 (6)
O7—Ca1—O5	74.02 (9)	O4B—C1—P1^i^	111.5 (2)
O7^iv^—Ca1—O5	138.18 (9)	O4A—C1—P1^i^	112.3 (2)
O3—Ca1—O5	119.24 (8)	C2B—C1—P1^i^	101.6 (3)
O3^iv^—Ca1—O5	69.84 (8)	C2A—C1—P1^i^	104.3 (2)
O6^v^—Ca1—O5	82.30 (7)	O4B—C1—P1	111.5 (2)
O6—Ca1—O5	68.18 (6)	O4A—C1—P1	112.3 (2)
O7—Ca1—O5^iv^	138.18 (9)	C2B—C1—P1	101.6 (3)
O7^iv^—Ca1—O5^iv^	74.02 (9)	C2A—C1—P1	104.3 (2)
O3—Ca1—O5^iv^	69.84 (8)	P1^i^—C1—P1	111.1 (2)
O3^iv^—Ca1—O5^iv^	119.24 (8)		

Symmetry codes: (i) *x*, *y*, −*z*; (ii) −*x*+*y*+1, −*x*+1, −*z*; (iii) −*x*+*y*+1, −*x*+1, *z*; (iv) *y*, *x*, −*z*+1/2; (v) −*y*+1, −*x*+1, −*z*+1/2; (vi) −*x*+1, −*y*+1, *z*; (vii) −*y*+1, *x*−*y*, *z*.

### Hydrogen-bond geometry (Å, º)

**Table:**

*D*—H···*A*	*D*—H	H···*A*	*D*···*A*	*D*—H···*A*
O5—H1···O1^viii^	0.84 (1)	1.95 (2)	2.756 (3)	160 (4)
O5—H2···O5^v^	0.79 (2)	2.07 (2)	2.799 (5)	155 (4)
O6—H3···O2^vi^	0.82 (2)	1.87 (2)	2.685 (3)	170 (4)
O7—H4···O3^viii^	0.82 (2)	2.07 (2)	2.865 (4)	166 (4)
O4*A*—H4*A*···O8	0.82	1.99	2.731 (16)	150

Symmetry codes: (v) −*y*+1, −*x*+1, −*z*+1/2; (vi) −*x*+1, −*y*+1, *z*; (viii) *x*−*y*, *x*, *z*.
